# The Expenditure on Vaccine Therapeutics

**Published:** 1912-12-07

**Authors:** 


### PROBLEMS IN ADMINISTRATIVE MEDICINE.

(Carefully thought-out Contributions to this Section will be welcome.)

### The Expenditure on Vaccine Therapeutics.

BY A PATHOLOGIST.

A STATEMENT that the principles of vaccine therapeutics require a thorough revision would be news to many who pride themselves on being up to date, but those who are acquainted with the history of its development and with its present condition would probably acquiesce. A review of the matter is a very grave commentary on the intellectual powers of a learned profession.

Vaccine therapy was introduced by Koch in the year 1890. It was entirely on empirical data and without any dependence on a "theory of immunity" that he introduced tuberculin. In spite of Koch's warnings there was a great "boom," and both doctors and patients flocked to Berlin. All kinds of unsuitable cases were injected, with the natural result—many fatalities. Then there was an irrational panic, and results due to bad technique and bad selection of cases, and last, but not least, to influenza, were attributed to the drug itself. In spite of a few almost miraculous cures which showed the wise that there was some good in the thing if they could only discover how to use it, the profession as a whole followed their leaders and dropped tuberculin like a hot brick. A few set out to re-examine the case and have reaped their reward, for again the whole profession seems to be converted. Tuberculin is being adopted by Governments and municipalities as one of the weapons to be used against tuberculosis. The thoughtful man, however, sees further dangers ahead. A review of current literature shows only too plainly that there is almost absolute divergence of view as to the selection of cases, that the modifications of tuberculin are almost endless in number, and that the system of dosage is also subject to many differences of opinion. Instead of these problems being carefully studied by highly competent students in hospitals the tuberculin treatment of tuberculosis has been handed over to numerous men, many of whom have had very little clinical experience, which exercises them in judging therapeutic results—such experience is hard to gain outside a large hospital. These men are paid officials, and they will have to produce statistics. The only way to get good results is to get "early" cases. Now a review of the statistics on the early diagnosis of tuberculosis shows a very unsatisfactory state of affairs. One famous expert, examining school children, places the number of tuberculous children at an alarmingly high figure, whilst a second, and equally competent man, gives an exceedingly low estimate. Examination by physical signs alone is absolutely unsatisfactory, as might be expected from such figures, yet specialists are crying out for diagnosis before bacilli are found.

In some quarters there is a great inclination to fly to tuberculin for diagnostic purposes. When it is realised that about 70 per cent. of healthy people give a positive tuberculin reaction owing to a small focus of *healed* tuberculosis, it is easy to see how an uncritical, although honest, man in search of early cases will find them. Those who have had most experience of tuberculin must regard this wholesale inoculation as a most disastrous course. Instead of being practised in hospitals under the critical eyes of colleagues, residents, and students, it is to be practised behind closed doors by paid officials.

It is a curious irony of fate that the present vogue of tuberculin is largely due to the advocacy of tuberculin by those who condemned the present dosage. Tuberculin was re-introduced into England by Sir Almroth Wright, and from a comparison of the Wright doctrines it is easy to see that the growth of vaccine therapy into the tuberculin dispensaries of the present day is not a growth of knowledge, but merely a change of vogue. Scientific criticism has been in abeyance. The experimental work which initiated the era of vaccine therapy forms practically the only scientific work which has been done on the subject. By means of the "Opsonic Index" Wright claimed to have shown that in cases of *localised* infections there is a diminished opsonic power in the serum of the patient infected; that by means of a correct dose of vaccine this opsonic power can be raised, with the accompanying phenomena of negative and positive phase; that with this rise in opsonic power the patient tends to recover, but that if the dose be too large there is a bad effect, and the opsonic power is diminished (excessive negative phase). If the dose of vaccine be too small there is no effect, therefore the dose must be regulated by the estimation of the "Opsonic Index." Finally, he said, for efficient action the vaccine must be an autogenous one and derived from the patient's own strain of bacilli. Whilst there can be no doubt but that Wright's conception of applying bacteriological methods to clinical medicine was one of the greatest conceptions of our generation, these propositions should have been examined critically.

There are two factors on which the doctrines of the Wright School depend for proof. One is the accuracy of the "Opsonic Index." The other is the numerous cures which have taken place whilst the patient has been under vaccine treatment. Neither of these will stand criticism. The "Opsonic Index" was attacked by Strangeways and his colleagues at Cambridge, and a few others in this country. The New York Board of Health in a very business-like manner set six workers to test the accuracy of the index. Their report was against the reliability of the index. The Johns Hopkins Laboratory also impugned the accuracy of Wright's methods. They were followed by a few workers in this country, but the vast majority applied vaccine treatment without any further attempt to valuate the methods. Wright's critics were answered by the statement made or implied that their technique was at fault, and further work from the Wright School introduced new methods of obtaining accuracy and attributed the failure of opponents to neglect of these precautions. *It is important to note that these later details were quite unmentioned in Wright's original papers*, and that therefore the foundations of the opsonic theory were undermined by the highest authority. The index now appears to have been almost universally abandoned. Either it is hopelessly inaccurate, and the fundamental doctrines of vaccine therapy are worthless, or it is of value, in which case a method capable of estimating the immunity of the patient—the most valuable clinical method ever invented—should at any cost be used for all serious cases. In the opinion of many clinicians its claims will not stand analysis. In the first place there is the everlasting question, *Post hoc* or *propter hoc?* In infections this is always most difficult to gauge. Thus the present writer has three times been urged by experienced surgeons to undertake vaccine treatment because the patient would certainly die if treated by ordinary methods. Through a little delay in the preparation of the vaccine the patient has recovered before vaccine could be injected. This objection can be met by the statement that the cures are vouched for by some eminent clinical observers, and therefore this argument cannot be used against vaccines.

There are, however, differences of opinion between the advocates of vaccine therapy which show how critically their statements must be regarded. There are first the differences of opinion as to the diseases which benefit by vaccine treatment. One observer obtained most excellent results in colon bacilluria, another found only temporary alleviation (only to be expected in the untreated course of the condition), whilst the urine continued to be infected. Some obtained good results in colitis, whilst others failed entirely. The same applies to many other conditions. Again there is the question of dosage. From the original statements of Wright one would have expected that the dosage would be limited to a very narrow range, but on examination of the literature one finds a most amazing range. Tuberculin was given by Wright in small infrequent doses (say, $\frac{1}{10000}$th of a milligram) about once in fourteen days, and he definitely stated that it was not necessary to increase the dose. At the present day tuberculin is given about twice a week in doses ranging up to one milligram. Practically no experimental work on animals has been performed either to explain the difference between these two systems or to determine which of them is the better. There has been no increase of knowledge but merely a change of fashion, which must be attributed to a tacit confession that the Wright system of giving tuberculin has not fulfilled expectations. In the case of gonococcal infections there are the same enormous differences in the dosage. One school gets good results with doses ranging from 2½ to 10 millions, whilst another school advise doses ranging from 250 to 1,000 millions. Surely a drug which permits such elasticity of dosage must be a very inactive one. Even in the case of ocular diseases, where an excessive focal reaction would be followed by very serious results, we believe that doses of 50 million are advised by reliable authorities.

The fact of the matter seems to be that, to obtain reliable clinical evidence, vaccine treatment should be systematically investigated on some disease of known ætiology and fairly uniform course, such as typhoid fever. The number of cases should be large, and, as nursing is such a large factor in success, alternate cases only should be inoculated. Puerperal fever, with its uncertain course, has afforded many triumphs (?) for vaccine therapy, whilst typhoid has apparently failed to give such startling success. Is not one justified in attributing this difference to the fact that one disease is full of surprises, whilst the other is much more uniform? When the treatment of diseases of known causation is in such an uncertain condition it is mere waste of time to deal with the school of therapeutic adventurers who treat rheumatoid arthritis, which is possibly due to oral and nasal sepsis by the isolation of one out of the many organisms which inhabit the nose and mouth, and which is possibly the cause of the lesions in the joints, and then give doses of vaccine from the organism which may or may not be optimum doses. The patient may possibly improve and this improvement may possibly be due to the treatment. The same applies to the vaccine treatment of dyspepsia, diabetes, and neurasthenia.

From the above it will be evident that the case for vaccine treatment is, on paper, a bad one, but probably all will admit that there is a germ of truth—of extremely valuable truth—if one could only obtain exact information. It should be possible to get such information by a collective inquiry. We should propose first of all to submit the "Opsonic Index" to an impartial test. The Local Government Board subsidise work on immunity by means of opsonic determinations. Cannot the slides used in these investigations be handed over to some impartial official who would re-number them and hand them over to a second opsonist—or even the first—for re-counting? The two sets of figures could then be collated and the standard of error worked out. Typhoid fever might be investigated on an organised plan designed to prevent error, and then finally a collective hospital inquiry might result in the average course of the different types of gonococcal rheumatism being worked out and compared with the course under different methods of treatment. Almost every physician believed in the value of the alkali treatment of acute rheumatism until Gull and Sutton showed the virtues of mint-water.

The question of the value of vaccine treatment is of great importance. If it is useless it is time that a stop was put to the vast expenditure on it. If valuable it should be used in the Poor-Law infirmaries to save the ratepayers' pockets, and as the data are accessible it seems to be quite within the province of the Local Government Board to spend a little money in learning the truth.

